# Multiplex determination of serological signatures in the sera of colorectal cancer patients using hydrogel biochips

**DOI:** 10.1002/cam4.692

**Published:** 2016-03-19

**Authors:** Veronika I. Butvilovskaya, Sofya B. Popletaeva, Vladimir R. Chechetkin, Zhanna I. Zubtsova, Marya V. Tsybulskaya, Larisa O. Samokhina, Leonid I. Vinnitskii, Aligeydar A. Ragimov, Elena I. Pozharitskaya, Galina A. Grigor´eva, Natalya Y. Meshalkina, Svetlana V. Golysheva, Nadezhda V. Shilova, Nicolai V. Bovin, Aleksander S. Zasedatelev, Alla Y. Rubina

**Affiliations:** ^1^ Engelhardt Institute of Molecular Biology (IMB RAS), Russian Academy of SciencesMoscowRussia; ^2^ Moscow Institute of Physics and Technology (State University)MoscowRussia; ^3^ V. M. Petrovsky Russian Center of Science and Surgery, Russian Academy of Medical SciencesMoscowRussia; ^4^ Moscow City Clinical Hospital No. 12Russia; ^5^ I.M. Sechenov First Moscow State Medical UniversityMoscowRussia; ^6^ Shemyakin‐Ovchinnikov Institute of Bioorganic Chemistry, Russian Academy of SciencesMoscowRussia

**Keywords:** Colorectal cancer, hydrogel biochips, tumor antibodies, tumor markers, tumor‐associated glycans

## Abstract

Colorectal cancer (CRC) is the third most common malignancy in industrialized countries. Despite the advances in diagnostics and development of new drugs, the 5‐year survival remains only 60–65%. Our approach to early diagnostics of CRC is based on the determination of serological signatures with an array of hemispherical hydrogel cells containing immobilized proteins and oligosaccharides (glycochip). The compounds immobilized on the glycochip include tumor‐associated glycans (SiaTn, Tn, TF, Le^C^, Le^Y^, SiaLe^A^, and Man*β*1‐4GlcNAc*β*) and antibodies against human immunoglobulins IgG, IgA, and IgM. The glycochip detects antibodies against tumor‐associated glycans in patients’ sera. The simultaneous measurement of the levels of immunoglobulins enhances the diagnostic impact of the signatures. In this work, we found previously unreported increase in antibodies against oligosaccharide Man*β*1‐4GlcNAc*β* in patients with CRC. In parallel with these experiments, we determined the levels of oncomarkers carcinoembryonic antigen (CEA), cancer antigen (CA) 19–9, CA 125, CA 15–3, human chorionic gonadotropin (HCG), and alpha‐fetoprotein (AFP) using another gel‐based biochip with immobilized antibodies (oncochip) developed earlier in our laboratory. In total, 69 samples from healthy donors, 33 from patients with colorectal carcinoma, and 27 from patients with inflammatory bowel diseases were studied. The use of combined signatures of antiglycan antibodies and oncomarkers provides much better predictive value than the conventional measurement of oncomarkers CEA and CA 19–9. Positive predictive value of CRC diagnoses using together glycochip and oncochip reached 95% with the sensitivity and specificity 88% and 98%, respectively. Thus, the combination of antibody profiling with detection of conventional oncomarkers proved to be a promising tool in diagnostics of CRC.

## Introduction

The incidence of colorectal cancer (CRC) in industrialized countries varies between 42 cases per 100,000 per year in the USA and 89 in Japan and is among the top two to four malignancies, depending on the country. The rate of mortality is also very high and varies from 10% of all cancer‐related deaths in the USA to about 13% in Japan. In the Russian Federation, these numbers are 42 and 14%, correspondingly (http://www.who.int/cancer/country-profiles/en/). Major risk factors for CRC are inflammatory bowel factors such as Crohn's disease (CD), ulcerative colitis (inflammatory bowel disease [IBD]), and diverticulitis. At the early stages, CRC develops with minimal clinical symptoms. At the same time, the mortality caused by CRC is considered one of the most preventable by early diagnostics.

Despite the recent advances in the development of biotechnological drugs, chemotherapy regimens, and diagnostic equipment, 5‐year survival of CRC patients remains low. In industrialized countries it varies from 58% in France to 68% in Israel. In the Russian Federation, it is 41% for colon cancer and only 30% for rectal cancer 1.

Screening tests of CRC are divided into cancer prevention and cancer detection tests. Cancer prevention tests are preferred over detection tests. Cancer prevention tests have the potential to image both cancer and polyps, whereas cancer detection tests have low sensitivity for polyps and typically lower sensitivity for cancer compared with that in cancer prevention tests (imaging tests).

Currently, cancer prevention tests of CRC employ rather complex, expensive, and sometimes invasive techniques: colonoscopy, lower gastrointestinal series (barium enema), computerized tomography colonography, flexible sigmoidoscopy. Colonoscopy every 10 years, beginning at age 50, remains the preferred CRC screening strategy 2. However, not all eligible persons are willing to undergo colonoscopy for screening purposes. For example, in the USA where the technology and procedure are widely available, the colorectal screening participation is still low among average‐risk adults in the range 29.8–55.2%^3^. As such, a noninvasive biomarker for the early detection of CRC remains a priority.

Tests that primarily detect cancer include both guaiac‐based fecal occult blood testing (FOBT) and immunochemical‐based FOBT (FIT) 4. These are not specific tests for CRC markers, and if found positive, the presence of CRC still must confirmed by a direct structural exam such as colonoscopy or imaging procedures 5.

In recent decades most research has been directed toward identification of DNA markers in stool 6. Many DNA mutations occurring in CRC have been described. These mutations include early events in tumor genesis, such as APC and K‐ras mutations, as well as later events, such as p53 and BAT‐26 mutations. The use of stool DNA as a marker for CRC has long been an actively explored idea. Most of these studies demonstrated that in asymptomatic persons at average risk for colorectal cancer multitarget stool DNA testing detected significantly more cancers than did fecal immunochemical test, but at the same time produced more false‐positive results 7.

On August 11, 2014, FDA approved the first noninvasive DNA screening test for colorectal cancer Cologuard (http://www.fda.gov/) that detects hemoglobin in a stool sample. Cologuard also detects certain mutations associated with colorectal cancer in the DNA of cells shed by advanced adenomas as stool moves through the large intestine and rectum. So far the approval of Cologuard did not change current practice guidelines for colorectal cancer screening. Stool DNA testing is not currently recommended by the United States Preventive Services Task Force (USPSTF) as a screening method for colorectal cancer. Among other guidelines, the USPSTF recommends adults age 50–75 at average risk for colon cancer to be screened using fecal occult blood testing, sigmoidoscopy, or colonoscopy.

It would be highly advantageous to develop an alternative modality based on blood biomarkers as the first‐line screening test. This will allow for the identification of high‐risk individuals among the general population. It is likely that serum markers for CRC cancer screening would be better accepted and achieve higher utilization rate than stool‐based and endoscopic tests.

Therefore, it is desirable to identify serum biomarkers or serum signatures that are capable of predicting high‐risk patients. Carcinoembryonic antigen (CEA) has been the only biomarker recommended by the American Society of Clinical Oncology and the European Group on Tumor Markers for use in the postoperative period for the early detection of recurrent or metastatic CRC 8. However, the level of CEA is not a reliable enough test for initial screening for colorectal cancer because of the large numbers of false‐positive and false‐negative reports 9.

Preoperative CEA and CA 19–9 levels have been used in the past as prognostic indicators in colorectal cancer. It remains unclear whether monitoring tumor marker CA 19–9 has any clinical benefit in the management of colorectal cancer patients 10. CA 19–9 level markedly increases in patients with adenocarcinomas of the gastrointestinal tract and is only slightly increased in inflammatory diseases, gastric ulcer, and pancreatitis. Elevated serum CA 19–9 has been found in patients with various gastrointestinal malignancies, especially pancreatic cancer 11, 12.

The percentage of correctly classified cases (PCCC) for CRC diagnostics based on combined determination of CEA and CA 19–9 depends on the methods of statistical processing of the results, the presence of comorbidity, and on the stage of CRC as defined by TNM classification. As a result, the range of diagnostic specificity of the combination of CEA and CA 19–9 reported in the literature appears to be very broad – from 18% to 65% 13, 14.

For improvement of CRC diagnostics and its applicability to early screening, multiplex detection of a panel of tumor marker proteins in serum (protein signature) is being used. Such panels usually include CEA, CA 19–9, CA 125, CA 15–3, human chorionic gonadotropin (HCG), alpha‐fetoprotein (AFP), and other tumor markers 15, 16. However, no combination reported so far offers sufficient statistical validity for clinical applications.

Gastrointestinal cancer is accompanied by overexpression of glycoprotein tumor markers with aberrant glycosylation pattern 17, 18. Such an aberrant glycosylation of components of cell membranes occurs due to the changes in the content or activity of several glycosyltransferases in cancer cells 19, 20. As the result, glycoproteins and glycosphingolipids modified with new glycans appear on the surface of cancer cells membranes 21. Thus, the structure of several dozens of onco‐associated glycans is known for CRC, cancers of breast, liver, ovaries, and prostate 22, 23, 24. In particular, it is known that in malignant cells of gastrointestinal tract CEA contains an increased amount of glycans terminated with Lewis^X^ (Le^X^) and Lewis^Y^ (Le^Y^) oligosaccharides 25. Another tetrasaccharide SiaLe^A^ is a specific determinant of antigen CA 19–9 26.

Neoantigens – aberrantly glycosylated proteins of malignant cells – induce corresponding autoantibodies and/or change the level of existing (natural) antibodies. One example of such a change is the correlation between the expression of T and Tn antigens and corresponding serum antibodies with prognosis in patients with carcinoma 27. Detection of autoantibodies to tumor antigens is a promising direction in the development of cancer diagnostics, because these antibodies can be detected in serum at the early stage of the disease 28, 29. We hypothesized that simultaneous detection of antibodies against tumor‐associated glycans in combination with multiplex measurement of several tumor markers in patients’ serum could be significantly superior in early diagnostics of CRC as compared with the detection of just two tumor markers, CA 19–9 and CEA.

The common tool for such multiplex analyses of individual samples is a biological microchip (biochip). In most cases, the microchips are designed as 2D‐microarrays, that is the probes (oligonucleotides, peptides, proteins, or glycans) are immobilized on the surface of plastic, chemically modified glass, or membrane support. Several versions of 2D protein microarrays were described for simultaneous detection of several cancer markers in patients’ serum 15, 30, 31. Using one of these microarrays, the so‐called biochip C12, serum samples of patients with CRC were tested for the presence of several tumor markers, but the resulting protein signatures failed to detect early forms of the disease 32.

Along with the development of protein microarray technology, several groups began to work on microchips with immobilized glycans (glycochips) 33, 34. Glycochips, or printed glycan arrays, are used for the detection of antiglycan antibodies (AGA) in patients’ serum, in particular, for ovarian cancer diagnostics and patients’ stratification 34, 35.

Our approach to diagnostics of CRC is based on the determination of serological signatures in blood of CRC patients using a combination of a microarray of hemispherical hydrogel cells containing immobilized antibodies specific to CEA, CA 19–9, CA 125, CA 15–3, HCG, and AFP (oncochip) and a glycochip containing immobilized glycans specific to antiglycan antibodies associated with CRC and antibodies against human immunoglobulins IgG, IgA, and IgM. As taking blood samples is a routine procedure, our technique can be conveniently applied to the clinical analysis. The principal possibility to perform high sensitivity quantitative immunoassays on glycochips with 3D hemispherical hydrogel cells on glass support and their general characteristics were described earlier 36. Here, we detected the levels of antibodies to glycans SiaTn, TF, Tn, Le^C^, Le^Y^, SiaLe^A^, and Man*β*1‐4GlcNAc*β* as well as the level of the immunoglobulins IgG, IgA, and IgM using glycochip. Concentrations of protein cancer markers CEA, CA 19–9, CA 125, CA 15–3, HCG, and AFP were determined by immunofluorescence analysis using a protein biochip diagnostic test system 37. The 3D hydrogel biochips were manufactured using previously described technology^38^ that yields gel cells of uniform size and distribution of the probes within the cells, which is necessary for quantitative analysis. The analysis combining antiglycan antibodies, tumor markers, and the level of the immunoglobulins IgG, IgA, and IgM has not been used previously in cancer diagnostics and proved to be promising.

## Materials and Methods

### Clinical samples

Serum samples from healthy donors (HD) were obtained from the Department of Transfusion of B.V. Petrovsky Russian Scientific Center for Surgery (Moscow). Serum samples from patients diagnosed with CRC were obtained from Petrovsky Russian Scientific Center for Surgery before any treatment. Serum samples from patients with IBD and CD were received from Sechenov First Moscow State Medical University. The sample information of each group, such as gender, ages, and number of subjects can be found in Table 1. The diagnoses were confirmed by independent methods: sigmoidoscopy or colonoscopy, pathology, and measurement of CEA and CA 19–9 in serum using ELISA system (Abbott Laboratories, Abbott Park, IL).

**Table 1:** Characteristics of patients who provided clinical samples

CRC sera (sera of patients with colorectal cancer)				
	Male: Mean age 59.3 years		Female: Mean age 58.3 years	
Gender	Rectum cancer, Stage I–IV	Colon cancer, Stage I–III	Rectum cancer, Stage I–III	Colon cancer, Stage I–III
Cases: Total, 33	12	8	10	3
Age				
>70	1	1	–	–
60–70	4	5	1	2
50–60	7	2	9	1

Healthy donors		
Gender	Male: Mean age 60.0 years	Female: Mean age 59.8 years
Cases: Total, 67	50	17
Age		
>70	2	1
60–70	8	10
50–60	40	6

IBD Sera (sera of patients with Crohn's disease and ulcerative colitis)				
	Male: Mean age 51.2 years		Female: Mean age 44.5	
Gender	Crohn's disease	Ulcerative colitis	Crohn's disease	Ulcerative colitis
Cases: Total, 27	8	2	10	7
Age				
>70	–	–	–	–
60–70	2	–	1	2
30–60	6	2	9	5

IBD, inflammatory bowel disease.

Venous blood (12 mL) was collected into vacutainer tubes “Improvacuter” (Improve Medical, Guangzhou, China), stored for up to 24 h at +4°C, and centrifuged at 3000*g* for 15 min at +4°C. Supernatants were immediately used for the analysis on biochips.

### Equipment and materials

To perform analyses on glycochip, the following synthetic glycans with aminospacers were used: Tn, SiaTn, TF, Le^C^, Le^Y^, SiaLe^A^, and Man*β*1‐4GlcNAc*β* (Lectinity Holdings, Moscow, Russia). Rabbit antibodies against human IgG, IgM, and IgA (RAH‐Iss) and their biotinylated conjugates (RAH‐Iss‐biot) were purchased from Imtek LLC (Moscow, Russia). Streptavidin and fluorescent dye Cy5 were purchased from GE Healthcare Bio‐Sciences (Pittsburgh, PA).

Hydrogel biochips were manufactured on glass micro slides Corning 2947 (Corning Inc., Corning, NY). Polyvinyl alcohol (PVA), MW 50 kD; polyvinylpyrrolidone (PVP), MW 360 kDa; Tween‐20; and G‐25 Sefadex^®^ coarse were purchased from Sigma‐Aldrich (St. Louis, MO); Bind Silane – from GE Healthcare Bio‐Sciences; and Micro Bio‐Spin chromatography columns – from Bio‐Rad Laboratories (Hercules, CA).

Immunofluorescence assays on microchips were carried out in plastic chamber with a volume of 120 *μ*L securely attached to glass supporting plate.

The following solutions were used: PBS (0.01mol/L Na‐phosphate buffer, pH 7.2, 0.15mol/L NaCl); washing solution PBST (PBS with 0.01% Tween‐20); blocking solution PBSP (PBS with 1% PVA), dilution buffer for immunoassays DB (PBS with 0.15% PVA and 0.15% PVP).

### Manufacturing of 3D hydrogel biochips

The biochips were manufactured by copolymerization immobilization 39. The polymerization mixture contained gel‐forming methacrylamide‐based monomers together with proteins or oligosaccharides intended for immobilization and modified with proper spacers. The mixture was applied in 0.1 nL microdroplets on Bind Silane‐activated microslides using a QArray robot («Genetix»Limited, New Milton, United Kingdom).

The polymerization of the gel cells was initiated by UV irradiation. After the completion of polymerization, microarrays were washed for 40 min in PBST, then in distilled water, and dried. The diameter of the resulting hemispherical 3D gel elements was 120 *μ*m.

Glycochips contained gel cells with glycans immobilized via spacers at a final concentration 3.3 mmol/L (total content 3.3 × 10^−13^ moles per cell). They are listed in Table 1. In addition, the glycochip contained cells with immobilized RAH‐Iss at 0.16 mg/mL (total amount 1.6 × 10^−11^ mg per gel element) and empty gel elements for nonspecific binding control. Each gel element was repeated four times, and the median value of four measurements was calculated as the final result.

To reduce non‐specific interactions, the biochips were incubated in PBSP for 1 h, then washed with distilled water and dried. The quality of the biochips was checked by their imaging in transmitted light in a dedicated biochip analyzer using software TestChip and QualityControl. Biochips were rejected when the deviation of the radii of gel elements exceeded 5% within individual biochips and 8% among the biochips of a single batch.

Ready‐to‐use biochips were covered with plastic chambers 120 *μ*L in volume with two openings for injecting a sample and stored at 2–8°C.

### Immunoanalysis on glycochips

Serum samples were diluted fivefold with DB, then 120 *μ*L of diluted serum applied on the chip and incubated for 2 h at 37°C. After an intermediate washing for 20 min in PBST, the chip was rinsed with distilled water and dried in a stream of air. Then 120 *μ*L of RAH‐Iss‐biot antibodies (0.01 mg/mL) were applied on the chip and incubated for 1 h at 37°C. The chip was washed in PBST for 20 min, rinsed with distilled water, and dried in a stream of air. Then 120 *μ*L of streptavidin conjugate with Cy5 (0.01 mg/mL) was applied on the chip and incubated for 10 min at 37°C. Final washing was performed in PBST for 30 min. The biochip was dried in a stream of air, and fluorescent signals were recorded using a GenePix 4200A scanner and GenePix 6.0 Pro software (both – Molecular Devices LLC, Sunnyvale, CA) for processing fluorescence images. The median feature pixel intensity of fluorescence at 635 nm (F635) was used for further calculations and interpretation of the results.

### Fluorescent immunoassay measurements

The signals obtained on glycochips were determined as follows:

(1) I=I(probe)−I(gel)

where *I*(probe) is fluorescent signal from the cells containing immobilized glycans or antibodies and *I*(gel) is fluorescent signal from blank cells, both signals in relative fluorescence units.

### Statistical analysis and data presentation

In evidence‐based medicine, the prognostic capability of a test system is often evaluated by the analysis of ROC‐curves (Receiver Operating Characteristic curve analysis) 40. This method allows comparing the efficacy of different diagnostic systems in detecting pathologies. To draw ROC‐curves, true‐positive rates are plotted against false‐positive rates obtained in the course of the study.

Comparative analysis of diagnostic significance of different factors in multiplex measurements on biochips was performed using multiparameter log‐regression analysis^41^ and ROC‐curves. The higher the area under the ROC‐curve (Area Under Curve, [AUC]), the higher the diagnostic efficacy of the corresponding factor. The diagnostic method is considered excellent when AUC = 0.9–1.0; very good at AUC = 0.8–0.9; good at AUC = 0.7–0.8; satisfactory at AUC = 0.6–0.7; and unsatisfactory at AUC = 0.5–0.6. In fact, AUC = 0.5 corresponds to random diagnostic outcome.

For quantitative assessment of the efficacy of diagnostics, two cohorts of patients are selected: without the pathological condition of interest (“healthy donors”) and with the condition diagnosed by unrelated means. The basic principle of the ROC analysis is the comparison between the sensitivity (*Se*) and specificity (*Sp*) of the model calculated from the number of false‐positive diagnoses. The calculations of Se and Sp operate not with absolute values, but with relative rates expressed as percentages. Thus, the rate of true‐positive results (True‐Positive Rates, TPR) is:

(2) $$TPR=\frac{TP}{TP+FN}100\text{percent}$$

where as the rate of false‐positive results (false‐positive rates, FPR) is:

(3) $$FPR=\frac{FP}{FP+TN}100\text{percent}$$

where *TP* (true positives) are correctly assigned positive results (correct positive diagnoses);

*TN* (true negatives) are correctly assigned negative results (correct negative diagnoses);

*FN* (false negatives) are positive results incorrectly assigned as negative (error of the first type). These are the so‐called “false failures to diagnose,” when an existing condition of interest (malignant disease) is not found;

*FP* (false positives) are negative results incorrectly assigned as positive (error of the second type). These are the so‐called “false findings of the condition,” when the condition of interest (malignant disease) is reported to be found while in fact it is absent.

Using ROC analysis, *Se* and *Sp* are calculated from the binary model using the following formulas. The sensitivity of the model is the proportion of *TP* results:

(4) $$Se=TPR=\frac{TP}{TP+FN}100\text{percent}$$

The specificity of the model is the proportion of *TN* results:

(5) $$SP=100\text{percent}-FPR=\frac{TN}{TN+FP}100\text{percent}$$

In the binary model of diagnostics, *Se* and *Sp* approach 100% (or AUC approaches unity), when the number of *FN* and *FP* results approaches zero.

Statistical processing of the results (concentrations of tumor markers, level of antibodies to glycans, and level of immunoglobulins calculated from fluorescence intensity after immunoassays on the biochips) was carried out using software MedCalc Version 11.4.2.0 (Ostend, Belgium) and Statistica Version 8.0 (StatSoft, Tulsa, OK). We used the following models:

1. The cohort of patients with diagnosis CRC and the cohorts of patients with IBD and of healthy donors (model “Oncology”, CRC = 1, IBD = 0, HD = 0);
2. The cohort of patients with gastrointestinal pathologies (CRC and inflammatory conditions) and the cohort of healthy donors (model “Disease”, CRC = 1, IBD = 1, HD = 0);
3. The cohort of patients with diagnoses CRC and the cohort of patients with IBD (model “CRC/IBD”, CRC = 1, IBD = 0).

Such an approach to classification of the diagnoses enabled us: (1) to identify patients with CRC using the “Oncology” model; (2) to differentiate all patients with gastrointestinal conditions from healthy donors using the “Disease” model; (3) and to differentiate CRC cases from inflammatory conditions using the “CRC/IBD” model.

## Results

### Measurements of antiglycan antibodies in serum samples on glycochip

**Figure 1:**
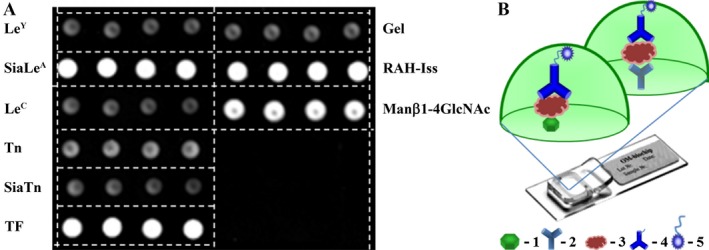
Analysis of sera samples on glycochip. A, Fluorescent image of glycochip after serum analysis obtained using the GenePix Pro 6.0 software. B, Scheme of assay on glycochip: 1 – immobilized glycan; 2 – immobilized antibody against human immunoglobulins IgG, IgA, and IgM; 3 – detected components of human serum; 4 – biotinylated antibodies against human immunoglobulins; 5 – Cy5‐labeled streptavidin.

Detection of antibodies against glycans SiaTn, Tn, TF, Le^C^, Le^Y^, SiaLe^A^, and Man*β*1‐4GlcNAc*β* (see Table S1 for their detailed nomenclature) was carried out on glycochips using sandwich immunofluorescent assay (Fig. 1). The chips were incubated with the samples of sera and then developed with biotinylated anti‐human antibodies and Cy5‐conjugated streptavidin.

The values of AUC (Table 2, “Oncology” model) for each of the seven glycans in detecting CRC revealed the signature consisting of the four antibodies to glycans, “set of four”, namely antibodies to Tn, TF, SiaLe^A^, and Man*β*1‐4ClcNAc, which has good predictive mutual capacity (AUC = 0.747). The most effective glycan to determine the CRC was Man*β*1‐4GlcNAc*β* (AUC = 0.725). The corresponding ROC curves for the resulting data can be found in Figure S2.

**Table 2:** ROC analysis of CRC diagnostic efficacy based on the immunofluorescent detection of antibodies to glycans using glycochips

Glycans	All	Set of four	Tn	Le^C^	Le^Y^	TF	SiaTn	SiaLe^A^	Man*β*1‐4GlcNAc*β*
“Oncology” model (CRC = 1, IBD = 0, HD = 0)									
AUC	0.883	0.747	0.616	0.503	0.509	0.605	0.564	0.700	0.725
Se (%)	45.5	36.4	21.2	12.1	15.2	21.2	6.1	27.3	33.3
Sp (%)	95.8	96.9	96.9	100.0	99.0	96.9	99.0	97.9	96.9
PCCC (%)	83.0	81.4	77.5	77.5	77.5	77.5	75.2	79.8	80.6
“Disease” model (CRC = 1, IBD = 1, HD = 0)									
AUC	0.914	0.596	0.524	0.699	0.617	0.547	0.643	0.587	0.509
Se (%)	80.0	28.3	26.7	5.0	15.0	26.7	0.0	26.7	21.7
Sp (%)	88.4	87.0	87.0	100.0	94.2	88.4	100.0	89.9	91.3
PCCC (%)	84.5	59.7	58.9	55.8	57.4	59.7	53.5	60.5	58.9
“CRC/IBD” model (CRC = 1, IBD = 0)									
AUC	0.915	0.851	0.640	0.736	0.643	0.614	0.773	0.721	0.826
Se (%)	84.9	69.7	54.6	54.6	45.5	48.5	54.6	63.6	66.7
Sp (%)	77.8	66.7	77.8	70.4	59.3	70.4	85.2	81.5	77.8
PCCC (%)	81.7	68.3	65.0	61.7	51.7	58.3	68.3	71.7	71.7
“Oncology” model (CRC = 1, IBD = 0, HD = 0); glycans and IgG+IgA+IgM									
AUC	0.959	0.956	0.847	0.806	0.780	0.834	0.807	0.921	0.948
Se (%)	84.9	84.9	42.4	18.2	24.2	27.3	12.1	75.8	81.8
Sp (%)	94.8	95.8	93.8	93.8	93.8	96.9	96.9	95.8	95.8
PCCC (%)	92.3	93.0	80.6	74.4	76.0	79.1	75.2	90.7	92.3
“Disease” model (CRC = 1, IBD = 1, HD = 0); glycans and IgG+IgA+IgM									
AUC	0.931	0.865	0.822	0.712	0.731	0.817	0.740	0.842	0.824
Se (%)	85.0	70.0	61.7	58.3	61.7	66.7	56.7	68.3	65.0
Sp (%)	89.9	82.6	85.5	72.5	75.4	78.3	73.9	78.3	78.3
PCCC (%)	87.6	76.7	74.4	65.9	69.0	72.9	65.9	73.6	72.1
“CRC/IBD” model (CRC = 1, IBD = 0); glycans and IgG+IgA+IgM									
AUC	0.953	0.932	0.772	0.817	0.727	0.763	0.857	0.883	0.924
Se (%)	90.9	90.9	78.8	87.9	75.8	66.7	81.8	87.9	81.8
Sp (%)	85.2	81.5	74.1	70.4	59.3	74.1	77.8	85.2	81.5
PCCC (%)	88.3	86.7	76.7	80.0	68.3	70.0	80.0	86.7	81.7

CRC, colorectal cancer; IBD, inflammatory bowel disease; HD, healthy donors; AUC, Area Under Curve; PCCC, percentage of correctly classified cases; Se, sensitivity; Sp, specificity.

The set of four glycans includes Tn, TF, SiaLe^A^, and Man*β*1‐4GlcNAc*β*. The larger the value of AUC, the higher the diagnostic efficacy of a method.

### The level of the immunoglobulins IgG, IgA, and IgM

In addition to immobilized glycans, glycochips contained cells with antibodies against human immunoglobulins making it possible to simultaneously measure the level of AGA against seven glycans and the level of the immunoglobulins IgG, IgA, and IgM in a single sample of serum (Fig. 1).

It is known that the concentration of immunoglobulins in the serum of healthy donors varies depending on age and sex 42. There are few reports on the immune status of CRC patients 43. Gough determined the level of IgG, IgA, and IgM in the serum of CRC patients and found an increase in levels of all three immunoglobulins in patients with relapsed CRC as compared to patients without recurrence 44.

**Figure 2:**
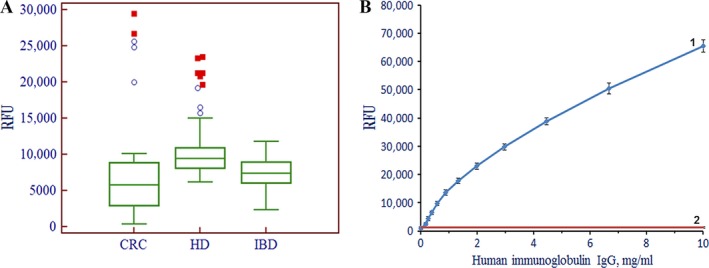
The level of immunoglobulins IgG, IgA, and IgM in the serum of patients. A, Box‐and‐whiskers charts for distributions of fluorescence signals obtained on glycochips from gel elements with immobilized antibodies to immunoglobulins IgG, IgA, and IgM. The chart HD corresponds to healthy donors; CRC to patients with colorectal cancer; and IBD to patients with irritable bowel syndrome. The boundaries of the box are the 25th and 75th percentiles, respectively; the line in the middle of the box corresponds to the median (50th percentile), whereas the mean value is marked by the square. The ends of the whiskers correspond to the edge of a statistically significant sample (no outliers). B, Fluorescence signal from gel elements with immobilized antibodies to IgG, IgA, and IgM versus IgG concentration in solution after development with biotin‐labeled secondary antibodies. The biochips were developed with Cy5‐conjugated streptavidin. 1 – Calibration curve for on‐chip sandwich immunoassay of human immunoglobulin IgG; 2 – Background signal from gel elements without immobilized antibodies. Each point of the calibration curve is the mean of measurements over ten biochips. HD, healthy donors; IBD, inflammatory bowel disease; CRC, colorectal cancer.

Figure 2A shows box‐and‐whiskers diagram of the results of immunofluorescent sandwich analysis of sera from 69 healthy donors, 33 CRC patients, and 27 patients with IBD. The measurements were carried out on glycochips with additional cells for the detection of three immunoglobulins IgG, IgA, and IgM. The median values for the level of three immunoglobulins IgG, IgA, and IgM are a little higher in CRC and IBD cohorts in comparison with that in HD cohort. The distribution of concentrations of immunoglobulins was highly variable within each cohort, and this variability was even more pronounced among the patients with CRC. Figure 2B demonstrates the possibility of determining concentration of human immunoglobulin G in serum on the biochip by calibration curve. For immunoassays in this work, we used the strongly nonequilibrium kinetic regime for immunoglobulin binding and development to shorten the time of analysis. As is seen from Figure 2B, kinetic regime permits to cover the dynamic range of immunoglobulin concentrations broader than 0.1–10 mg/mL. The comparison of data in Figures 2A and B proves that the range of immunoglobulin concentrations 0.1–10 mg/mL is of practical importance.

**Figure 3:**
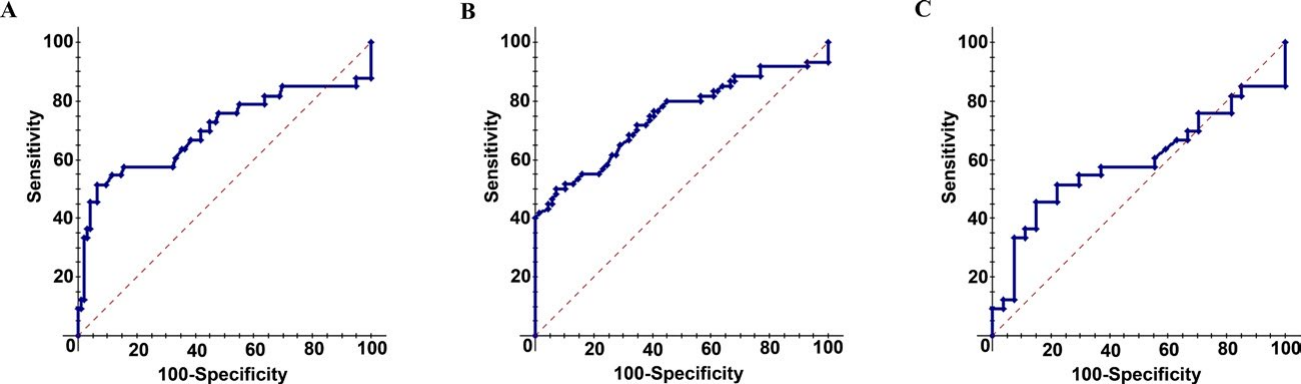
ROC‐curves for level of three immunoglobulins IgG, IgA, and IgM as diagnostic criterion. A, “Oncology” model, AUC = 0.663; B, “Disease” model, AUC = 0.747; C, “CRC/IBD” model, AUC = 0.590 (see “Statistical processing and data presentation”). ROC‐curves, Receiver Operating Characteristic curve analysis; AUC, Area Under Curve; CRC, colorectal cancer; IBD, inflammatory bowel disease.

As turned out, the level of the immunoglobulins IgG, IgA, and IgM in the serum is in itself a significant prognostic factor. Specifically, in the model “Oncology” the value of AUC was 0.663, in the model “Disease” AUC = 0.747, and in the model “CRC/IBD” the level of immunoglobulins is noninformative (Fig. 3).

As seen from the second part of Table 2, the addition of data on immunoglobulins IgG, IgA, and IgM to data on AGA significantly improves the CRC diagnostic efficacy. In particular, the addition of data on immunoglobulins IgG, IgA, and IgM to data on the signature consisting of the combination of four glycans Tn, TF, SiaLe^A^, and Man*β*1‐4GlcNAc*β* increases the number of correctly classified diagnoses from 81% to 93%. If the level of antibodies to Man*β*1‐4GlcNAc*β* is considered apart, the addition of the data on nonspecific immunoglobulin levels increases the sensitivity of CRC detection from 33% to 82%, whereas the PCCC of correct diagnoses increases from 81% to 92%.

### Measurements of protein tumor markers in serum samples on oncochip

Detection of tumor markers AFP, HCG, CEA, CA 19–9, CA 125, and CA 15–3 in serum samples was performed on protein biochip by immunofluorescence analysis as described earlier 37. The study of 33 samples from the patients with CRC revealed increased concentration of CEA in 17% of patients, CA 19–9 in 13%, CA 125 in 17%, and CA 15–3 in 13%. The level of HCG was increased in one patient, whereas the level of AFP was within the normal range in all patients. Although the common tumor markers CEA and CA 19–9 reveal high specificity in CRC diagnostics, their sensitivity turns out to be rather low. The same is true for the whole set of oncomarkers (Table 3, “Oncology” model).

**Table 3:** ROC analysis of CRC diagnostic efficacy based on the immunofluorescent detection of antibodies to glycans using glycochips together with the immunofluorescent detection of oncomarker levels using oncochips

Markers	All glycans and oncomarkers	Four glycans and oncomarkers	Oncomarkers	CEA and CA 19–9
“Oncology” model (CRC = 1, IBD = 0, HD = 0)				
AUC	0.915	0.854	0.817	0.594
Se (%)	54.6	48.5	27.3	21.2
Sp (%)	94.8	95.8	97.9	100.0
PCCC (%)	84.5	83.7	79.8	79.8
“Disease” model (CRC = 1, IBD = 1, HD = 0)				
AUC	0.956	0.867	0.847	0.670
Se (%)	83.3	66.7	66.7	48.3
Sp (%)	91.3	91.3	92.8	87.0
PCCC (%)	87.6	79.8	80.6	69.0
“CRC/IBD” model (CRC = 1, IBD = 0)				
AUC	0.974	0.902	0.793	0.684
Se (%)	90.9	84.9	75.8	78.8
Sp (%)	88.9	81.5	55.6	51.9
PCCC (%)	90.0	83.3	66.7	66.7
“Oncology” model (CRC = 1, IBD = 0, HD = 0); oncomarkers, glycans, and IgG+IgA+IgM				
AUC	0.972	0.962	0.841	0.761
Se (%)	87.9	81.8	48.5	27.3
Sp (%)	97.9	96.9	94.8	96.9
PCCC (%)	95.4	93.0	83.0	79.1
“Disease” model (CRC = 1, IBD = 1, HD = 0); oncomarkers, glycans, and IgG+IgA+IgM				
AUC	0.977	0.964	0.900	0.830
Se (%)	88.3	83.3	71.7	68.3
Sp (%)	91.3	89.9	91.3	82.6
PCCC (%)	89.9	86.8	82.2	76.0
“CRC/IBD” model (CRC = 1, IBD = 0); oncomarkers, glycans, and IgG+IgA+IgM				
AUC	0.991	0.954	0.796	0.685
Se (%)	97.0	90.9	75.8	72.7
Sp (%)	96.3	88.9	59.3	51.9
PCCC (%)	96.7	90.0	68.3	63.3

CEA, carcinoembryonic antigen; CA, cancer antigen; CRC, colorectal cancer; IBD, inflammatory bowel disease; HD, healthy donors; AUC, Area Under Curve; Se, sensitivity; Sp, specificity; PCCC, percentage of correctly classified cases.

The set of oncomarkers includes CEA, CA 19–9, CA 125, CA 15–3, human chorionic gonadotropin, and AFP, whereas the set of four glycans includes Tn, TF, SiaLe^A^, and Man*β*1‐4GlcNAc*β*. The larger the value of AUC, the higher the diagnostic efficacy of a method.

### Comparison of multiplex determination of protein and antiglycan signatures for detection of CRC

Comparative analysis of the diagnostic efficiency of protein and glycan signatures and their combinations performed in multiplex format on biochips was carried out using multiple log‐regression analysis^41^ and ROC‐curves 45. The larger the area under the ROC‐curve (AUC), the higher the diagnostic efficiency of a given signature. The values of the following diagnostic parameters were used for such an analysis: concentrations of tumor markers CEA and CA 19–9 (these are the oncomarkers used in conventional detection of CRC), CA 125, CA 15–3, HCG, and AFP; the level of AGA against glycans Tn, TF, SiaLe^A^, and Man*β*1‐4ClcNAc (henceforth referred as “set of four glycans”) as well as Le^C^, Le^Y^, and SiaTn; and the level of the immunoglobulins IgG, IgA, and IgM in serum. The results of statistical processing of the combined data obtained in the simultaneous measurement of tumor markers, antibodies to glycans, and the level of the total immunoglobulins IgG, IgA, and IgM in serum are summarized in Table 3.

## Discussion

Current colorectal cancer screening modalities are inadequate for mass application because of high costs and a low participation rate. Ideally, a blood‐based test could be used to identify high‐risk patients, who would then be examined by colonoscopy as a secondary test.

The monitoring of specific tumor marker levels in sera has become a conventional clinical technique in diagnostics of certain malignancies, whereas the efficacy of the monitoring of AGA using glycan probes in oncology has been recognized rather recently. The efficiency of CRC diagnostics using most known tumor markers appears to be rather low (see, e.g., the data for the conventional pair CEA and CA 19–9 in Table 3). Equally low is the diagnostic efficiency of individual glycans (Table 2). Unlike tumor markers, the addition of the total level of immunoglobulins IgG, IgA, and IgM as a diagnostic parameter strongly improves the diagnostic efficacy of some glycans. In particular, we found a novel glycan CRC marker Man*β*1‐4GlcNAc*β*, which, together with the level of IgG, IgA, and IgM, provides both high sensitivity *Se* = 81.8% and specificity *Sp* = 95.8%.

The multiplex analysis based on a specific set of different parameters proved to be significantly more reliable in diagnosing CRC than any individual parameter. We found that adding the level of three immunoglobulin IgG, IgA, and IgM as a diagnostic parameter strongly improves the diagnostic efficiency of both particular probes and the set of probes (Tables 2 and 3). Combining glycans, tumor markers, and the level of the immunoglobulins IgG, IgA, and IgM allowed us to attain PCCC = 95%, *Se* = 88%, and *Sp* = 98% in CRC diagnostics as compared with PCCC = 80%, *Se* = 21%, and *Sp* = 100% using just CEA and CA 19–9.

Neither glycans (SiaTn, Tn, TF, Le^C^, Le^Y^, SiaLe^A^, and Man*β*1‐4GlcNAc*β*), nor tumor markers (CEA, CA19–9, CA 125, CA 15–3) used in this study have been previously reported to be specific to CRC. Nevertheless, the combined multiplex analysis including these signatures turned out to be rather efficient for CRC diagnostics. The ability of these serological signatures to discriminate between CRC and other tumors remains still an open question that requires a large‐scale study with multiple types of tumors and other clinical attributions. We intend to verify this ability in subsequent investigations.

The state‐of‐the‐art manufacturing permits to produce microarrays with tens to thousands cells containing different probes. However, the increase in the number of parameters per set would not necessarily lead to better diagnostic efficacy; on the contrary, addition of irrelevant noninformative probes may act as a noise and deteriorate the diagnostic efficiency. Besides, the problem of cost cannot be neglected at the mass manufacturing of microarrays for clinical applications. Our results provide clear evidence that using relatively restricted set of molecular markers could be quite efficient in CRC diagnostics. Although in this work we used protein biochips and glycochips as separate tools, further development of microarray technology will enable us to combine these arrays into a single diagnostic device.

It should be noted that the reproducibility and analytical sensitivity of the technology of 3D hydrogel‐based microarrays are comparable to ELISA, whereas the analytical dynamic range is considerably higher 38, 46. This may favor practical application of multiplex hydrogel‐based biochips in the diagnosis of colorectal cancer and other malignancies.

## Conflict of Interest

None declared.

## Supporting information


**Table S1.** The list of oligosaccharides immobilized in gel cells via spacers

**Figure S2.** ROC analysis of the results obtained for serum autoantibodies against individual tumor‐specific glycans, model «Oncology» (see “Statistical analysis and data presentation”).
