# Supplementary material for: Multiplex determination of serological signatures in the sera of colorectal cancer patients using hydrogel biochips
Source: Cancer Med. 2016 Mar 19;5(7):1361–72. doi: 10.1002/cam4.692 (PMC4944861; doi:10.1002/cam4.692)
Supplement: Supplementary file 1 — Table S1. The list of oligosaccharides immobilized in gel cells via spacers [file CAM4-5-1361-s001.doc]

**Table S1**. The list of oligosaccharides immobilized in gel cells via spacers

| No. | Spacered oligosaccharides | Common name | Molecular  weight, a.u. |
| --- | --- | --- | --- |
| 1 | GalNAcα-sp3 | Tn | 278.3 |
| 2 | Neu5Acα2-6GalNAcα-sp3 | SiaTn | 569.6 |
| 3 | Fucα1-4  GlcNAcβ-sp3  Neu5Acα2-3Galβ1-3 | SiaLe^A^ | 877.9 |
| 4 | Galβ1-3GlcNAcβ-sp3 | Le^C^ | 426.4 |
| 5 | Fucα1-2Galβ1-4  GlcNAcβ-sp3  Fucα1-3 | Le^Y^ | 732.7 |
| 6 | Galβ1-3GalNAcα-sp3 | TF | 440.5 |
| 7 | Manβ1-4GlcNAcβ-sp3 | Manβ1-4GlcNAcβ | 439.4 |

sp3 = -O(CH_2_)_3_NH_2_

The total concentration of each glycan per gel element was 3.3×10^-13^ mole/spot.
