# Supplementary material for: Multiplex determination of serological signatures in the sera of colorectal cancer patients using hydrogel biochips
Source: Cancer Med. 2016 Mar 19;5(7):1361–72. doi: 10.1002/cam4.692 (PMC4944861; doi:10.1002/cam4.692)
Supplement: Supplementary file 2 — Figure S2. ROC analysis of the results obtained for serum autoantibodies against individual tumor‐specific glycans, model «Oncology» (see “Statistical analysis and data presentation”). [file CAM4-5-1361-s002.doc]

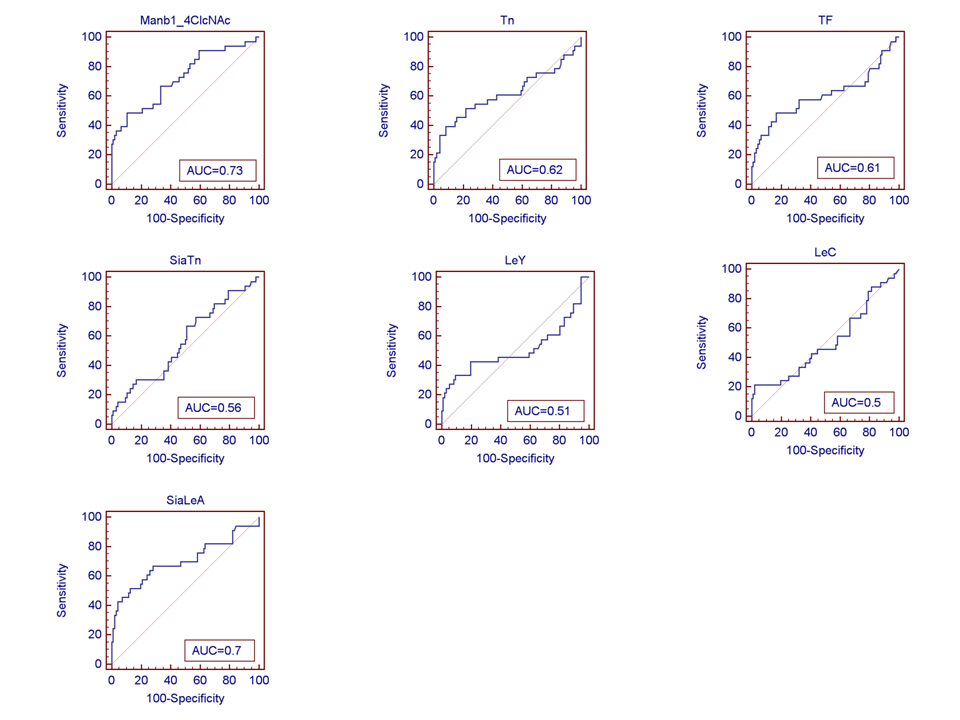


Fig. S2. ROC analysis of the results obtained for serum autoantibodies against individual tumor-specific glycans, model «Oncology» (see "Statistical analysis and data presentation").
